# Clinical research updates

**DOI:** 10.1111/camh.70053

**Published:** 2025-12-16

**Authors:** Marinos Kyriakopoulos, Anastasia Andreou, Dimitrios Apostolou, Paraskevi Lampropoulou

**Affiliations:** ^1^ National and Kapodistrian University of Athens Athens Greece; ^2^ Department of Child and Adolescent Psychiatry, Institute of Psychiatry Psychology and Neuroscience King's College London London UK; ^3^ South London and Maudsley NHS Foundation Trust London UK; ^4^ University of Ioannina Ioannina Greece

## 1 Predictors of adult psychiatric outcomes of childhood attention‐deficit/hyperactivity disorder

Anastasia Andreou

National and Kapodistrian University of Athens

Most individuals with childhood Attention‐Deficit/Hyperactivity Disorder (ADHD) experience remittance of their ADHD diagnosis over time, but impairing symptoms of ADHD may persist, and psychiatric disorders are highly prevalent in these individuals. There is limited understanding about childhood factors that may predict these outcomes.

van der Plas et al. (2025) conducted a systematic review and meta‐analysis aimed at identifying childhood factors that predict psychiatric problems in adults diagnosed with childhood ADHD. The final analysis included 36 longitudinal studies investigating 119 unique predictors, although a majority (66%) were studied only once. Meta‐analyses were conducted when at least three studies examined the same predictor (10 predictors) with comparable effect sizes; otherwise, narrative reviews were performed (31 predictors). Study quality was assessed using the Newcastle–Ottawa Scale (NOS), with only 44% of studies rated as fair or good quality. Additionally, 53% of studies did not control for confounding variables. Five predictors of ADHD persistence were meta‐analyzed. A childhood history of stimulant treatment was associated with an increased risk for persistent ADHD, although children with more severe symptoms may be more likely to receive these medications. Higher childhood Full Scale IQ was associated with a decreased risk, but this association lost significance when one poor‐quality study was excluded. ADHD persistence was linked to an increased risk of substance use disorders (SUDs) and major depressive disorder (MDD) in adulthood but not to adult anxiety disorders. Childhood oppositional defiant disorder/conduct disorder (ODD/CD), male sex, and severity of childhood ADHD‐related impairment were not associated with adult ADHD persistence. Childhood ODD/CD and stimulant treatment did not predict adult SUDs. Narratively reviewed predictors from fair/good quality studies showed some evidence that ADHD combined type, hyperactive/impulsive symptoms, anxiety disorders, externalizing problems, social dysfunction, and low socioeconomic status increase the risk of ADHD persistence. There was also some narrative evidence linking persistent ADHD to adult antisocial personality disorder (ASPD), late initiation of stimulant treatment to increased risk of SUDs in adulthood, and stimulant treatment generally to decreased risk of adult MDD. This study confirms many findings from previous meta‐analyses but highlights discrepancies regarding childhood IQ, ODD/CD, and severity of ADHD impairment. Unlike earlier work, childhood MDD was not found to predict adult ADHD persistence.

The authors emphasize the need for further investigation into predictors such as ADHD combined type, hyperactive/impulsive symptoms, anxiety disorders, externalizing problems, social dysfunction, thought problems, parental psychopathology, and socioeconomic status. They highlight limitations due to the number of available studies and the types of predictors and outcomes studied. Future research is recommended to include a broader range of potential predictors, utilize advanced methods such as machine learning, and apply individual participant data meta‐analyses to better understand combined predictor effects and rarer adult outcomes of childhood ADHD.

van der Plas, N.E., Noordermeer, S.D.S., Oosterlaan, J., & Luman, M. (2025). Systematic review and meta‐analysis: predictors of adult psychiatric outcomes of childhood attention‐deficit/hyperactivity disorder. *Journal of the American Academy of Child and Adolescent Psychiatry*: S0890‐8567(25)00215‐1. doi: 10.1016/j.jaac.2025.04.012.

## 2 Joint developmental trajectories of internalizing and externalizing problems from mid‐childhood to late adolescence and childhood risk factors

Dimitrios Apostolou

National and Kapodistrian University of Athens

Internalizing problems—such as anxiety, depression, social withdrawal, and somatic complaints—and externalizing problems—such as aggression, impulsivity, and conduct difficulties—typically emerge in early childhood and may persist into adolescence and adulthood, leading to adverse developmental outcomes. Although traditionally studied as separate pathways, a growing body of research indicates that internalizing and externalizing problems are not independent but frequently co‐occur and interact across development. This co‐occurrence is associated with greater psychosocial maladjustment, poorer academic outcomes, and increased risk of later psychopathology.

Bista et al. (2025) conducted a large prebirth cohort of approximately 2000 participants followed from birth to late adolescence. Using latent class growth analysis (LCGA), the authors identified subgroups of children with distinct co‐occurring trajectories of internalizing and externalizing problems based on repeated parent and teacher reports. The aim was to determine whether these two domains of psychopathology follow independent or interrelated developmental courses.

Four main trajectory groups emerged. The low‐stable class represented the majority of participants with consistently low symptom levels. The moderate‐decreasing group showed elevated difficulties in childhood that diminished over time. An increasing group exhibited a progressive rise in both internalizing and externalizing problems, while a high‐stable group maintained persistently high levels across all developmental stages. Roughly 8–10% of participants belonged to the two most symptomatic trajectories.

Several early risk factors differentiated these groups. Children exposed to maternal psychopathology and prenatal stress, lower socioeconomic status, inconsistent or harsh parenting, and early temperament dysregulation were significantly more likely to follow increasing or high‐stable trajectories. These findings support the concept of developmental comorbidity, where emotional and behavioral problems are intertwined and reinforce each other over time.

Strengths include the study's prospective design, multi‐informant repeated assessments, and use of advanced statistical modeling, which allow for nuanced depiction of developmental heterogeneity. However, limitations involve reliance on parental reports, potential attrition bias, and limited exploration of mediating biological or contextual mechanisms.

In conclusion, the study provides compelling evidence that internalizing and externalizing problems often co‐develop in identifiable patterns from childhood through adolescence. Early family and individual factors play a pivotal role in shaping these trajectories, underscoring the importance of long‐term monitoring and early, family‐focused prevention strategies in promoting child and adolescent mental health.

Bista, S., Tait, R. J., Straker, L. M., Lin, A., Steinbeck, K., Graham, P. L., … & Skinner, S. R. (2025). Joint developmental trajectories of internalizing and externalizing problems from mid‐childhood to late adolescence and childhood risk factors: Findings from a prospective pre‐birth cohort. *Development and Psychopathology*, 37(1), 176–191. doi: 10.1017/S0954579423001505.

## 3 Psychosis and bipolar disorder in individuals with attention‐deficit/hyperactivity disorder treated with stimulants

Paraskevi Lampropoulou

University of Ioannina

Stimulants are the first‐line medication treatment for Attention‐Deficit/Hyperactivity Disorder (ADHD). Despite the benefits they have in the core symptoms of ADHD, they have some adverse effects. It has been suggested that stimulants may induce psychotic and manic symptoms that can lead to a psychotic disorder or bipolar disorder.

Salazar de Pablo et al. (2025) performed a systematic review and meta‐analysis exploring the impact of stimulants on psychotic symptoms or mania/bipolar disorder symptoms in individuals who received these medications for ADHD. The search included databases such as PubMed, Web of Science, Ovid/PsycINFO, and Cochrane Central Register of Reviews, covering literature from inception until October 1, 2024. A total of 16 studies were included, comprising 391,043 individuals aged between 8.5 and 31.1 years (mean 12.6). Participants had a diagnosis of ADHD according to DSM or ICD and had reported bipolar disorder, psychosis, or symptoms of these conditions while they were under stimulant medication.

The meta‐analytical results indicated that 2.76% of individuals with ADHD who had been prescribed stimulants developed psychotic symptoms, 2.29% a psychotic disorder, and 3.72% bipolar disorder. Heterogeneity was statistically significant among included studies (*I* ^2^ > 95%) for both psychotic and bipolar disorder symptoms. According to the subgroup analysis, there were differences related to study continent and the follow‐up duration. Individuals who were prescribed amphetamines presented with more psychotic symptoms than those prescribed methylphenidate (57% higher odds). A higher rate of females, stimulant dose, and smaller sample size led to publication bias. In quality assessment, 71.4% of the cohort studies were rated as good. One RCT was evaluated as having low risk of bias and the other as having some concerns. The mechanisms that can trigger psychosis or bipolar disorder are not fully understood. Some individuals could present with such symptoms following stimulant medication, but this may not represent a cause–effect relationship.

Several limitations were identified. Firstly, the studies had no control group comparison (individuals treated with non‐stimulants) and only three studies compared amphetamines with methylphenidate. In addition, it was not possible to perform a meta‐analysis on data regarding the development of manic symptoms associated with stimulant use. Furthermore, despite exploring heterogeneity via meta‐regressions, comprehensive assessment was limited by the scarcity of reported data on ADHD severity, comorbidities, medication use, and psychosocial functioning. It was also not possible to explore age effects whilst the pattern of medication use and the significance of any baseline symptoms of psychosis or bipolar disorder could not be evaluated. Finally, some of the data were from electronic databases and therefore less accurate.

Further research on this field is warranted. Clinicians should inform their patients about their therapeutic plan and all the side effects, as stimulants may be associated with a clinically relevant risk of psychosis and bipolar disorder.

Salazar de Pablo, G., Aymerich, C., Chart‐Pascual, J.P., Solmi, M., Torres‐Cortes, J., Abdelhafez, N., … & Cortese, S. (2025). Occurrence of psychosis and bipolar disorder in individuals with attention‐deficit/hyperactivity disorder treated with stimulants: A systematic review and meta‐analysis. *JAMA Psychiatry*. doi: 10.1001/jamapsychiatry.2025.2311.

## 4 Conflict of interest statement

M.K. is the *CAMH* Associate Editor for Clinical Research Updates. The editor thanks the contributors for this issue's Clinical Research Updates. The editor has declared that he has no competing or potential conflicts of interest.

## 5 Ethics statement

No ethical approval was required for these updates.

## Data Availability

Data sharing is not applicable to this article as no new data were created or analyzed.

